# A Cycle Slip Detection and Repair Method Based on Inertial Aiding for BDS Triple-Frequency Signals

**DOI:** 10.3390/s23125641

**Published:** 2023-06-16

**Authors:** Xiyu Fu, Yongrong Sun, Ling Wu, Kaifeng Wang, Kedong Zhao

**Affiliations:** College of Automation Engineering, Nanjing University of Aeronautics & Astronautics, Nanjing 211106, China

**Keywords:** BeiDou navigation satellite system, inertial navigation system, tightly coupled integration, triple-frequency signals, cycle slip detection and repair

## Abstract

Cycle slip detection and repair is a prerequisite to obtain high-precision positioning based on a carrier phase. Traditional triple-frequency pseudorange and phase combination algorithm are highly sensitive to the pseudorange observation accuracy. To solve the problem, a cycle slip detection and repair algorithm based on inertial aiding for a BeiDou navigation satellite system (BDS) triple-frequency signal is proposed. To enhance the robustness, the INS-aided cycle slip detection model with double-differenced observations is derived. Then, the geometry-free phase combination is united to detect the insensitive cycle slip, and the optimal coefficient combination is selected. Furthermore, the L2-norm minimum principle is used to search and confirm the cycle slip repair value. To correct the INS error accumulated over time, the extended Kalman filter based on the BDS/INS tightly coupled system is established. The vehicular experiment is conducted to evaluate the performance of the proposed algorithm from a few aspects. The results indicate that the proposed algorithm can reliably detect and repair all cycle slips that occur in one cycle, including the small and insensitive cycle slips as well as the intensive and continuous cycle slips. Additionally, in signal-challenged environments, the cycle slips occurring 14 s after a satellite signal outage can be correctly detected and repaired.

## 1. Introduction

The real-time kinematic (RTK) technology based on differential carrier phase can achieve centimeter-level high-precision positioning. It has been used in many fields, such as surveying, mapping, navigation, and so on. However, the carrier phase cycle slip will occur in the case of low cut-off elevation angle, covered by high buildings, receiver failure, and high dynamic conditions [1,2,3]. Therefore, it is difficult to guarantee positioning accuracy.

The cycle slip detection methods commonly used contain the high-order difference method, ionosphere residual algorithm, TurboEdit algorithm, pseudorange and phase combination algorithm, geometry-free (GF) phase combination algorithm, second-order time-difference phase ionospheric residual (STPIR) algorithm, and so on [4,5]. However, they are not suitable to some respective cases. For example, it is difficult for the high-order difference method to detect small and continuous cycle slips. The ionosphere residual algorithm is insensitive to the cycle slip, which is equal to the ratio of dual frequency. The pseudorange and phase combination algorithm is restrained by the pseudorange observation accuracy [6]. Through continuous research, scholars have made a theoretical and practical significance in research findings. One way is to improve the algorithm to adapt to the conditions. Xie et al. proposed an improved high-order difference method based on double difference observation, which can effectively overcome the deficiency in the face of small cycle slips [7]. Elashiry et al. improved the phase-code differencing and phase–phase differencing methods. The least-square technique is used to estimate the change in the ionospheric values and the code bias, which succeeds in improving the detection precision [8]. Using the TurboEdit algorithm, it can be improved by considering MW (Melbourne–Wübbena), GF combination of the process and the detection threshold [9]. The MW method combined with the ionospheric residual method is widely used to detect cycle slip. Cai et al. developed a reconstruction doppler integral to replace the insufficient MW method [10]. The other way is to combine other methods together as a supplement. Xu et al. presented cycle slip detection and repair for the BeiDou triple-frequency signal with pseudorange-phase combination and GF phase combination [11]. Cederholm et al. proposed a method that uses expected Doppler shifts to predict double differenced carrier phase observations, which can effectively identify cycle slips [12]. Considering that the longer the wavelength, the easier it is to detect and repair, the cycle slip can be detected by linear combinations: extra-wide lane (EWL), wide lane (WL), and narrow lane (NL) [13,14]. The pseudorange-phase combination and GF phase combination can be united for the BeiDou triple-frequency cycle slip detection and repair. Some scholars have conducted research on the BeiDou triple-frequency pseudorange-phase combination and GF phase combination and selected the optimal coefficient combination to detect the cycle slip [15,16]. Recently, some scholars have made new attempts. Suzuki considered a factor graph structure that included cycle slip factors using time differences of carrier phase observations [17]. The experimental results show that the cycle slip can be properly detected, estimated, and corrected by graph optimization. Yoon et al. proposed a method that detects the cycle clip in multiple channels [18]. It is also possible when the same size cycle slip occurs simultaneously in different channels.

The integration of a global navigation satellite system (GNSS) and an inertial navigation system (INS) has been widely deployed due to their complementary characteristics. It can maintain navigation information output in the GNSS signal outage condition. Wang et al. proposed an INS-aided single-frequency cycle slip detection method and emphatically analyzed the error characteristic of the detection terms [19]. Lee et al. and Altmayer provided an effective cycle slip detection method for GPS/INS-integrated systems that use additional information provided by the INS [20,21]. Wang et al. derived the inertial aided three-difference cycle slip detection formula and emphatically proposed an inertial-aided sliding window detection method [22]. Kim et al. discussed the cycle slip detection algorithm with inertial sensors and amply derived the influence of INS errors, and they suggested a methodology for the optimal selection of inertial sensors [23,24]. According to the Kalman filter innovation test theory, Gu et al. researched the cycle slip detection method in a GNSS/INS tightly coupled system and effectively improved the performance of cycle slip detection [25]. Chen et al. proposed a novel cycle slip processing strategy based on the TDCP-GNSS/INS integration scheme, in which cycle slip is handled with a robust extended Kalman filter (EKF) [26]. Vanek et al. presented a prediction-based cycle slip detection method [27]. They used the predicted states of the navigation EKF in a single epoch to handle the possible cycle slips and designed the experiments to prove the superiority of the method. Takasu et al. proposed an INS/GPS integration scheme for INS-aided cycle slip detection and fixing, which improves the performance of mobile vehicle navigation in severe conditions, such as urban areas [28]. Further, with the availability of multi-frequency signals, many researchers focus on triple-frequency cycle slip detection and repair methods. In theory, triple-frequency signals can be capable of improving the ability to detect the cycle slips. Ning et al. established the INS-aided cycle slip detection monitoring value based on a BDS/INS loose-coupled model, which can effectively resist the low-accuracy pseudorange observations. It improved the success rate and repair rate in a multipath effect environment [29]. Han et al. developed an inertial-aided GPS cycle slip detection and repair method for a GPS/INS tightly coupled system and verified effectively when there was a GPS data outage [30]. Xiao et al. proposed an inertial-aided triple-frequency cycle slip detection and repair method and analyzed the statistical results of the missing detection rate and false detection rate [31]. Traditional methods suffer from problems, such as poor robustness, blind zone of detection, and susceptibility to errors. They may seriously be affected by pseudorange observation noise. Meanwhile, combined with the GF method, the condition number of the combination coefficient matrix may lead to the ill-conditioned equation problem [6,32]. Currently, there are few research papers that involve BDS/INS tightly coupled integration to the cycle slip detection process based on double-differenced observations. The BDS triple-frequency signal combined observations and tightly coupled integration can provide improved cycle slip detection performance, which can still maintain reliable repair capability even when satellite signals are unlocked.

In the article, we derived a cycle slip detection and repair algorithm based on inertial aiding using double-differenced observations. The INS-predicted value is constructed to replace the pseudorange and enhance the robustness. What is more, we unite the GF phase combination to detect the insensitive cycle slip. In the cycle slip repair, the L2-norm minimum principle is used to search and confirm it further. To correct the INS error accumulated over time, the extended Kalman filter based on the BDS/INS tightly coupled system is established. Meanwhile, the optimal coefficient combination is selected, and we analyzed the performance of the proposed cycle slip detection and repair method by vehicle experiment.

This article is organized as follows: Section 2 derives the model of a BDS/INS tightly coupled navigation system. The inertial-aided cycle slip detection and repair model for the BDS triple-frequency signal is elaborated in Section 3. In Section 4, the performance of the proposed algorithm is compared and discussed using the experimental data. Finally, in Section 5, we give some conclusions.

## 2. BDS/INS Tightly Coupled Integration Model

The inertial navigation system, which is characterized by considerable short-term high accuracy, a high update rate, and autonomy, is the most widely used autonomous navigation system. Therefore, the integration of GNSS and INS has been widely deployed due to their complementary characteristics. It can still obtain better short-term accuracy in signal-challenged environments. In this paper, the BDS and INS data are fused based on extended Kalman filter.

### 2.1. INS Dynamic Model

The system state-space model can be described by the INS error model and system error from inertial sensors [33,34]:

(1) $$\left\{ \begin{array}{l} \delta \dot{r}=-\omega_{en}\times \delta r+\delta v\\ \delta \dot{v}=-(2\omega_{ie}+\omega_{en})\times \delta v-\psi \times f+\delta g+\nabla \\ \dot{\psi }=-\left(\omega_{ie}+\omega_{en}\right)\times \psi -\varepsilon \end{array}\right.,$$

where δr, δv, and ψ represent the position, velocity, and attitude error vectors; ε and ∇ are the bias errors for gyroscopes and accelerometers; f and g denote the specific force and gravity vector; $\omega_{ie}$ is the earth rotation rate with respect to the inertial frame; and $\omega_{en}$ is the rotation rate of the navigation frame with respect to the earth frame, which is selected as earth-centered earth-fixed (ECEF) frame in this paper.

### 2.2. BDS/INS Measurement Model

The measurement model is determined by the carrier phase and pseudorange observation equations. In the case of short baseline, the satellite and receiver clock biases, ionospheric and tropospheric effects, and other minor correction terms are almost eliminated by using a double-differenced (DD) technique. It is important to be clear that the state vector should be converted to an ECEF frame. The measurement equations are written as follows [35,36]:

(2) $$\{\begin{array}{l} \lambda (\nabla \Delta \varphi -\nabla \Delta N)-\nabla \Delta \rho_{INS}=A\cdot C_{n}^{e}\cdot \delta r+\varepsilon_{\nabla \Delta \varphi }\\ \nabla \Delta P-\nabla \Delta \rho_{INS}=A\cdot C_{n}^{e}\cdot \delta r+\varepsilon_{\nabla \Delta P} \end{array},$$

where φ and P represent the carrier phase and pseudorange; N denotes the integer ambiguity; A is the design matrix; $C_{n}^{e}$ is the transformation matrix from navigation frame to earth frame; $\varepsilon_{\nabla \Delta }$ is the DD measurement noise; and $\rho_{INS}$ is the INS-predicted distance deduced as:

(3) $$\rho_{INS}=\left\|r^{s}-r_{INS}\right\|=\sqrt{{\left(x^{s}-x_{INS}\right)}^{2}+{\left(y^{s}-y_{INS}\right)}^{2}+{\left(z^{s}-z_{INS}\right)}^{2}},$$

where $\left(x^{s},y^{s},z^{s}\right)$ is the satellite position calculated by corresponding ephemeris, and $\left(x_{INS},y_{INS},z_{INS}\right)$ is the INS-predicted receiver position. As the lever-arm term is small, it has been omitted in Equation (2). However, it should be noted that the lever-arm effect needs to be corrected to compensate for the separation of IMU (inertial measurement unit) location and GNSS antenna center when they have long distances or high maneuverability [37].

### 2.3. Extended Kalman Filtering Model

Therefore, the state equation of the BDS/INS tightly coupled system can be expressed as:

(4) $$X_{k}=\Phi_{k,k-1}X_{k-1}+\Gamma_{k-1}W_{k-1},$$

where $X_{k}$ is the state vector; $\Phi_{k,k-1}$ is the state transition matrix; $\Gamma_{k-1}$ is the noise coefficient matrix; and $W_{k-1}$ is the system noise vector. In the tightly coupled GNSS/INS systems based on undifferenced measurements, it is essential to add the distance error equivalent to the clock error and the distance rate error equivalent to the clock frequency error to the state vector. Since the model in the paper is derived based on the DD measurements, the clock biases are not considered. Therefore, the state vector is designed as $X={\left[\begin{array}{lllll} \delta r^{T} & \delta v^{T} & \psi^{T} & \varepsilon^{T} & \nabla^{T} \end{array}\right]}^{T}$. The measurement equation of the BDS/INS tightly coupled system is:

(5) $$Z_{k}=H_{k}X_{k}+V_{k},$$

where $Z_{k}$ is the measurement vector, $H_{k}$ is the design matrix, and $V_{k}$ denotes the measurement noise matrix. The IMU error can be corrected through the filtering feedback loop. Meanwhile, we can get the filtering results. The form of the filtering equation has been mentioned in detail in corresponding papers [34,36], so they will not be repeated here redundantly.

## 3. Inertial-Aided Cycle Slip Detection and Repair for BDS Triple-Frequency Signals

### 3.1. Cycle Slip Detection Model Based on Inertial Aiding

In relative positioning, the carrier phase observations can be used to construct DD combination observations based on BDS triple-frequency B1, B2, and B3 signals:

(6) $$\nabla \Delta \varphi_{\left(i,j,k\right)}=\nabla \Delta \rho /\lambda_{\left(i,j,k\right)}+\nabla \Delta N_{\left(i,j,k\right)}+\varepsilon_{\nabla \Delta I}+\varepsilon_{\nabla \Delta \varphi }.$$

In Equation (6), there is

(7) $$\nabla \Delta \varphi_{\left(i,j,k\right)}=i\nabla \Delta \varphi_{1}+j\nabla \Delta \varphi_{2}+k\nabla \Delta \varphi_{3},$$

(8) $$\nabla \Delta N_{\left(i,j,k\right)}=i\nabla \Delta N_{1}+j\nabla \Delta N_{2}+k\nabla \Delta N_{3},$$

(9) $$\lambda_{\left(i,j,k\right)}=\frac{\lambda_{1}\lambda_{2}\lambda_{3}}{i\lambda_{2}\lambda_{3}+j\lambda_{1}\lambda_{3}+k\lambda_{1}\lambda_{2}},$$

(10) $$\varepsilon_{\nabla \Delta I}=-\eta_{\left(i,j,k\right)}\nabla \Delta I_{1},$$

(11) $$\eta_{\left(i,j,k\right)}=\frac{i}{\lambda_{1}}+\frac{\lambda_{2}}{\lambda_{1}^{2}}j+\frac{\lambda_{3}}{\lambda_{1}^{2}}k,$$

where i, j, and k are the carrier phase combination coefficients; 1, 2, and 3 represent the BDS frequencies; λ is the wavelength that corresponds with frequencies; $\varepsilon_{\nabla \Delta I}$ denotes the DD ionospheric residuals; $\varepsilon_{\nabla \Delta \varphi }$ is the DD carrier phase residual noise; $I_{1}$ is the ionospheric delay values at the first frequency; and η represents the ionospheric delay coefficient. Other symbols not mentioned are the same as the previous section.

In the traditional pseudorange and carrier phase combination method, the geometry-term is eliminated by differencing the carrier phase and pseudorange combination observation. However, the detection sensitivity is restricted by the pseudorange. It will be unreliable when pseudorange noise and multipath effect are serious. Consequently, take the INS predicted satellite-to-ground geometric distance into Equation (6):

(12) $$\nabla \Delta N_{\left(i,j,k\right)}=\nabla \Delta \varphi_{\left(i,j,k\right)}-\frac{\nabla \Delta \rho_{INS}}{\lambda_{\left(i,j,k\right)}}+\varepsilon_{\nabla \Delta I}+\varepsilon_{\nabla \Delta \varphi }+\varepsilon_{\nabla \Delta \rho },$$

where $\varepsilon_{\nabla \Delta \rho }$ represents the DD predicted distance error. Then, differencing Equation (12) between adjacent epochs t and t−1, which is deduced as follows:

(13) $$D_{1}=\nabla \Delta N_{\left(i,j,k\right)}^{t}-\nabla \Delta N_{\left(i,j,k\right)}^{t-1}.$$

In the case of a short baseline, the satellite and receiver clock biases and tropospheric effects have been eliminated. Therefore, the detection precision is determined by noise and wavelength of combination observation and the distance error that INS predicted. What is more, the ionospheric delay between adjacent epochs can be neglected under a 1Hz or higher sampling rate [15]. Assuming that each frequency of BDS has the same carrier phase noise error, the standard deviation of the cycle slip detection term can be calculated according to the error propagation law:

(14) $$\sigma_{D_{1}}=\sqrt{2}\sqrt{(i^{2}+j^{2}+k^{2})\sigma_{\nabla \Delta \varphi }^{2}+{\left(\frac{\sigma_{\nabla \Delta \rho_{INS}}}{\lambda_{\left(i,j,k\right)}}\right)}^{2}}.$$

In order to maintain higher sensitivity of detection term, we hope that it may have a smaller standard deviation and longer wavelength. Furthermore, the combination coefficients should be an integer. The variance of carrier phase can be written as $\sigma_{\varphi }^{2}=\sigma_{0}^{2}/sin^{2}(el)$, where el is the elevation angle, and $\sigma_{0}^{}$ is standard carrier phase noise selected as 0.01 cycle [38,39]. Some optimal triple-frequency pseudorange-phase combinations are calculated and listed in Table 1.

**Figure 1:**
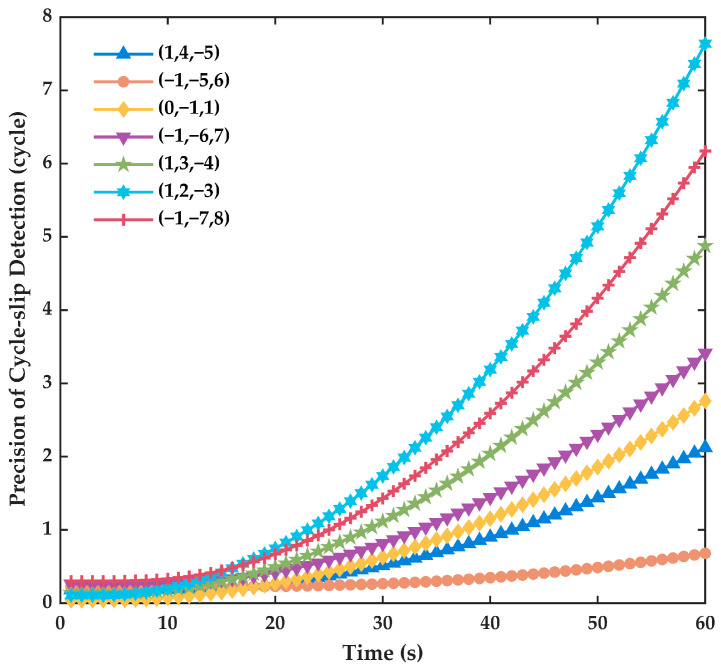
The precision impact on cycle slip detection when INS navigates independently.

**Figure 2:**
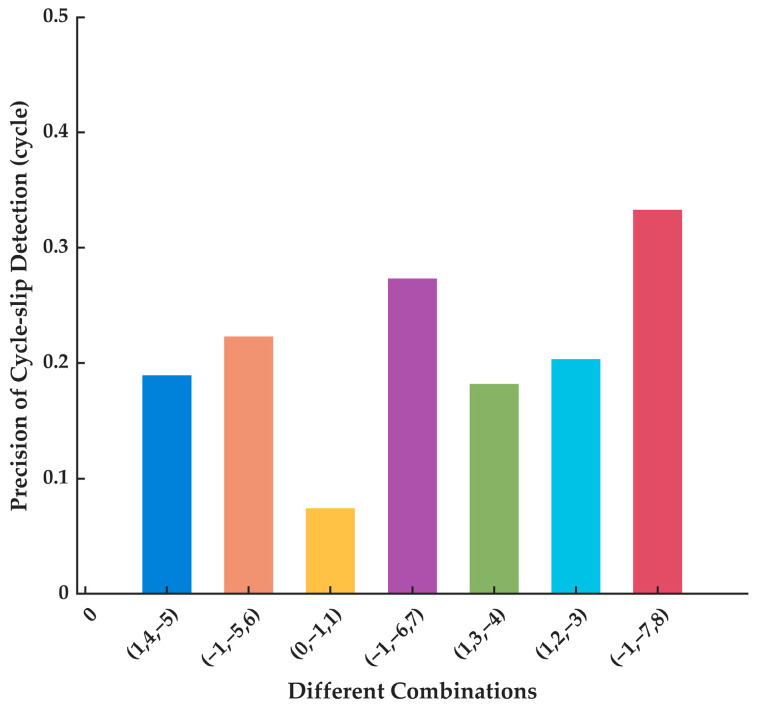
The precision of cycle slip detection for a 10 s signal outage.

The wavelength, ionospheric delay and carrier phase noise are taken into account to select the coefficient combinations. However, the i+j+k=±1 combinations have higher ionospheric delay, which will be harmful to the detection sensitivity of cycle slip. Therefore, the combination coefficients will be chosen from those that satisfy the condition i+j+k=0. From Equation (14), the precision of the detection term is correlated with the combination coefficients and INS errors. So, based on Equation (1) for error propagation calculations, we simulated the INS to further analyze the precision impact on cycle slip detection for different combinations in INS navigating independently, with an IMU whose gyroscope bias error is 1°/h and accelerometer bias error is 40 μg. The time for INS to navigate independently is 60 s. Actually, the influence of the INS uncertainty and their projection on DD needs to be analyzed theoretically from the full 3D case in further research. Figure 1 shows the precision impact on cycle slip detection when INS navigates independently. What is more, the precision of cycle slip detection for a 10 s signal outage is shown in Figure 2.

As the independent navigation time of INS prolongs, the precision of cycle slip detection decreases for each combination coefficient. The detection precision is related to the carrier phase observation noise if the signal is normal. At the same time, though the ionospheric delay can be ignored in the DD observation, the ionospheric delay should not be too large to affect the detection. Generally analyzing Figure 1 and Figure 2 and Table 1 in common, it can be discovered that the combination (0,−1,1) has a relatively smaller ionospheric delay and a longer wavelength. What is more important, it has the smallest combination noise. Although the precision decreases when INS navigates independently, the combination (0,−1,1) has a higher short-term navigation precision and obvious advantages. So, the combination (0,−1,1) is selected as the first combination coefficient. The divergence speed of (1,2,−3) is too fast to use, though its short-term navigation precision is higher. The combination (−1,−5,6) maintains a longer wavelength and a high detection accuracy of a 0.27 cycle within a 30 s signal that is unlocked, so it is chosen as the second coefficient combination.

### 3.2. Supplement for Particular Cases

The insensitive cycle slips are defined as the cycle slips that cannot be detected by the combinations. For the optimal combination coefficients selected in Section 3.1, if the cycle slip values are the same across all three frequencies, that is $\delta N_{1}=\delta N_{2}=\delta N_{3}=\delta N$, the detection terms will satisfy:

(15) $$\{\begin{array}{l} -\delta N_{2}+\delta N_{3}=0\\ -\delta N_{1}-5\delta N_{2}+6\delta N_{3}=0 \end{array}.$$

Apparently, the cycle slips cannot be identified, because it is the insensitive cycle slips for the two group coefficients. Additionally, solving three unknown cycle slips needs three equations, which are linearly independent. Therefore, another coefficient combination, which is linearly independent with above, is needed.

The geometry-free phase combination is not affected by the pseudorange noise or multipath, whose sensitivity is more significant [40]. Hence, it will be considered as a supplementary set. The coefficient of the combination observation geometry term is zero, that is a+b+c=0. Based on the DD observations, we can deduce a similar equation as:

(16) $$\begin{array}{l} B_{\left(a,b,c\right)}=a\lambda_{1}\nabla \Delta \varphi_{1}+b\lambda_{2}\nabla \Delta \varphi_{2}+c\lambda_{3}\nabla \Delta \varphi_{3}\\ \;\;\;=(a+b+c)\nabla \Delta \rho +\nabla \Delta N_{\left(a,b,c\right)}+\varepsilon_{\nabla \Delta I}+\varepsilon_{\nabla \Delta \varphi } \end{array}.$$

In Equation (16), there is

(17) $$\nabla \Delta N_{\left(a,b,c\right)}=a\lambda_{1}\nabla \Delta N_{1}+b\lambda_{2}\nabla \Delta N_{2}+c\lambda_{3}\nabla \Delta N_{3},$$

(18) $$\varepsilon_{\nabla \Delta I}=-\eta_{\left(a,b,c\right)}\nabla \Delta I_{1},$$

(19) $$\eta_{\left(a,b,c\right)}=a+b\frac{\lambda_{2}^{2}}{\lambda_{1}^{2}}+c\frac{\lambda_{3}^{2}}{\lambda_{1}^{2}},$$

Then, differencing Equation (17) between adjacent epochs, which is deduced as follows:

(20) $$D_{2}=\nabla \Delta N_{\left(a,b,c\right)}^{t}-\nabla \Delta N_{\left(a,b,c\right)}^{t-1}.$$

According to the error propagation law, the standard deviation of the cycle slip detection term is as follows:

(21) $$\sigma_{D_{2}}=\sqrt{2}\sqrt{(a^{2}\lambda_{1}^{2}+b^{2}\lambda_{2}^{2}+c^{2}\lambda_{3}^{2})\sigma_{\nabla \Delta \varphi }^{2}}.$$

The geometry-free phase combinations should satisfy the condition a+b+c=0. Additionally, the optimal combinations can be selected with the lower ionospheric delays and lower combination noises. According to the conditions above, the values of the coefficients a, b, and c are searched within the range of −5 to 5. In order to analyze the optimal combination, we can further compare the properties of different combination observations listed in Table 2.

When the three frequencies have the same cycle slips, the combinations (0,−1,1) and (−1,−5,6) are disabled. Hence, we should select the combination coefficients that have lower noises to prevent missing detection. The combination (1,−1,0) has the minimum standard deviation. At the same time, if the detection term satisfies $\left|D\right|<3\sigma_{D_{2}}=0.027$, the combination will not discover the cycle slips since we can solve that −0.473<δN<0.473. So, according to the analysis above, the insensitive cycle slips can be successfully detected in theory, whose value is equal to or greater than one cycle.

### 3.3. Methodology to Confirm and Repair Cycle Slip

According to the three independent linear combinations detected by analyzing above, the cycle slip can be confirmed and repaired. The cycle slip is considered to occur when the following condition is satisfied:

(22) $$\left|D\right|>k\sigma_{D}.$$

Generally, we take *k* = 3 and 4 as the threshold scale factor at the confidence level of 99.7% and 99.9% [11,16,29]. Three linearly independent combinations have been used in the cycle slip detection. When a cycle slip occurs at a certain epoch, the cycle slip value will be calculated according to the linearly independent equations as follows:

(23) AX=L,

where X is the original value of cycle slip; L is the cycle slip observation, and A represents the combination coefficient matrix: $A=\left[\begin{array}{ccc} i_{1} & j_{1} & k_{1}\\ i_{2} & j_{2} & k_{2}\\ a\lambda_{1} & b\lambda_{2} & c\lambda_{3} \end{array}\right]$.

As soon as the cycle slips are estimated, the triple-frequency raw carrier-phase data can be corrected by $\hat{X}={\left(A^{T}A\right)}^{-1}A^{T}L$, theoretically. Since A is non-singular, it can be simplified to $\hat{X}=A^{-1}L$. However, it could be ill-conditioned because of the condition number of A. Accordingly, when L is influenced by the observation noise, the estimated cycle slip value may be incorrect [3,38]. Avoiding the problems above and to ensure the success rate of cycle slip repair, we consider the methodology as follows: Search the value with a $k\sigma_{D_{2}}$ radius centered at the L. The L2-norm is used to judge if the cycle slip correction value $\hat{X}$ is correct, which should satisfy the condition [11,15]:

(24) $${\left\|A\hat{X}-L\right\|}_{2}=min.$$

That is, the sum of squares of the difference between $A\hat{X}$ and L is minimized, where $A\hat{X}$ represents the changes in the detection term caused by the estimated cycle slip $\hat{X}$. Hence, the correct original cycle slip can be guaranteed from the method mentioned.

## 4. Experimental Results and Discussion

**Figure 3:**
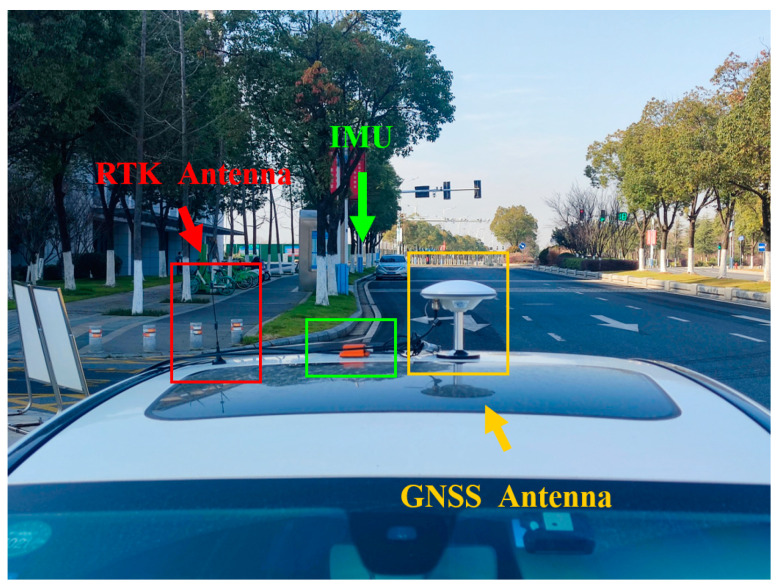
Experimental platform and hardware.

In order to test the performance of inertial-aided cycle slip detection and repair for triple-frequency signals, a vehicular experiment around Nanjing City in China was conducted. The experimental IMU is the MTI-G-700 inertial sensor produced by XSENS Co. (Enschede, The Netherlands), whose gyroscope in-run bias stability is 10°/h and accelerometer in-run bias stability is 40 μg. In addition, the NovAtel OEM7 multi-frequency receivers, whose sampling rate is 1 Hz, were used to collect GNSS measurement data. During the experiment, a total of approximately 33 min of data (2000 available epochs in total) was collected for postprocessing. In this paper, the GNSS data, which are used to verify the conclusion, are based on the BeiDou-3 global satellite navigation system (BDS-3). The experimental data have been preprocessed so that it does not include any cycle slips. Figure 3 shows the experimental platform and hardware.

**Figure 4:**
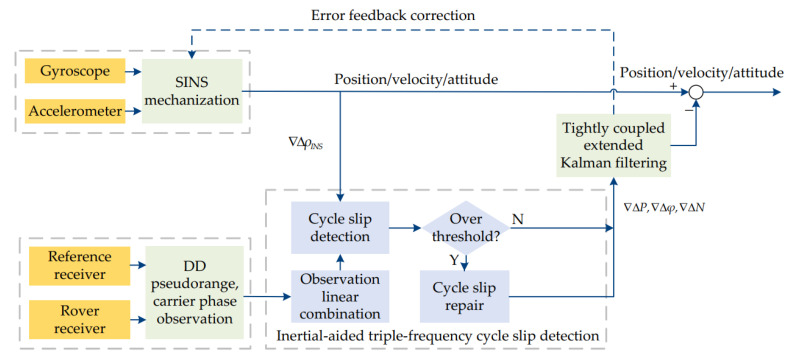
Inertial-aided cycle slip detection and repair based on the BDS/INS tightly coupled system.

Figure 4 shows the flowchart of the inertial-aided cycle slip detection and repair based on the BDS/INS tightly coupled system, which mainly includes: (1) IMU and SINS mechanization; (2) the DD observation equation founded and linear combination; (3) inertial-aided cycle slip detection and repair; (4) extended Kalman filter based on a tightly coupled system; and (5) IMU error feedback correction.

### 4.1. Performance Analysis of Cycle Slip Detection and Repair Based on Inertial Aiding for BDS Triple-Frequency Signals

All types of the BeiDou satellites (MEO, GEO, and IGSO) are contained in the observation data. Choosing Satellite C33 as the reference satellite, Table 3 shows the standard deviation (STD) of the detection terms for the traditional and improved algorithms when no cycle slip occurs. The corresponding satellite elevation angles are also included in the table.

**Figure 5:**
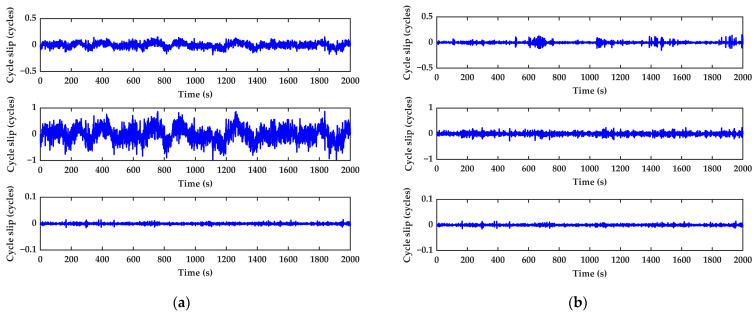
Different cycle slip detection models for C25 when no cycle slip occurs: (**a**) The traditional pseudorange and phase combination method; (**b**) INS-aided cycle slip detection and repair-improved method.

As can be seen from Table 3, the STD of the detection term for the method based on INS aiding is significantly reduced, leading to an improvement in the detection accuracy. The average standard deviation of the combination (0,−1,1) decreased from 0.0708 to 0.0422, while that of the (−1,−5,6) combination decreased from 0.3349 to 0.1021. Meanwhile, there is a certain correlation between the STD of the detection term and the elevation angle of the satellite. For satellites with low elevation angles, the STD of the cycle slip detection terms will increase. Taking satellite C25 as an example, Figure 5 shows the value of the satellite C25 cycle slip detection combinations. Respectively, the subfigures from top to bottom denote the combinations Nos. 1, 2, and 3.

It can be seen from Figure 5 that, constrained by the dynamic observation conditions, the traditional model can cause false detection. For the lower elevation angle satellites, the phenomenon will be more serious. The value of the combination (0,−1,1) fluctuates around ±0.2 cycle. At the same time, the combination (−1,−5,6) value approaches ±1, which may result in false alarm obviously. The geometry-free phase combination is not affected by the pseudorange observation noise. Summarizing the analysis above, the traditional model fails in the dynamic environment with lower pseudorange observation accuracy. In this case, the performance requirements cannot be met unless the robustness is improved. In Figure 5b, it can be found that when no cycle slip occurs the value is stable in general, fluctuating up and down near zero. Owing to the INS characterized by considerable short-term accuracy and analysis without using the pseudorange observation, the cycle slip detection value does not affect by pseudorange observation noise. Hence, it has a higher detection accuracy compared with the traditional pseudorange-phase combination algorithm.

**Figure 6:**
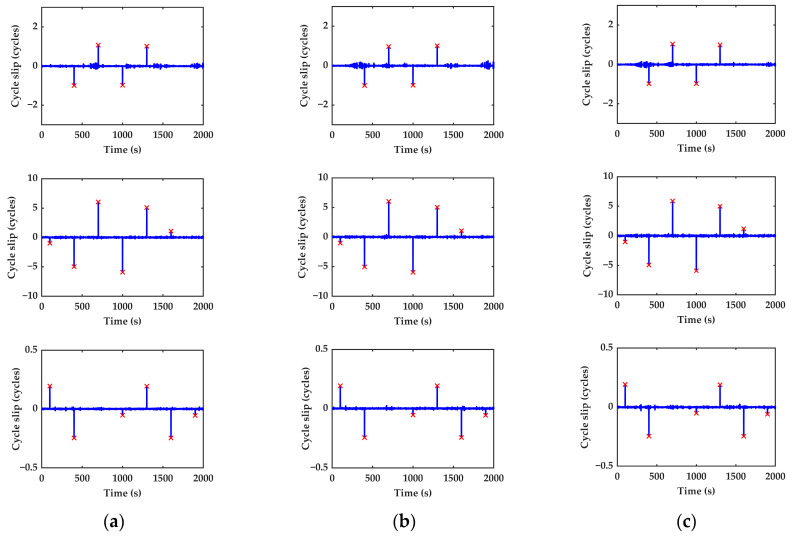
The detection results of the cycle slip simulation (Scheme I): (**a**) C25; (**b**) C39; (**c**) C59.

*Scheme I.* In order to verify the performance of the cycle slip detection and repair algorithm, different cycle slips, whose values are one cycle, are added in the BeiDou satellite C25 (MEO), C39 (IGSO), and C59 (GEO) different epochs of carrier-phase observations with an interval of 300 epochs. This covers all cases where the cycle slip value is equal to 1. Therefore, the one cycle small cycle slips are simulated at the first 1600th epochs, respectively. Combinations (1,0,0), (0,1,0), and (0,0,1) are the small, simulated cycle slips, which occur on a single frequency. The small cycle slips, which are insensitive to combination Nos. 1 and 2, are simulated at the 1900th epochs. Table 3 shows the value and location of simulated cycle slips. Generally, the cycle slip, whose value is one cycle, is the most serious condition for detection, and the missing detection occurs easily. If the algorithm should detect the cycle slips, it would also detect other bigger ones. The identified cycle slips are marked and indicated by “×”. The detection results of Scheme I are shown in Figure 6 and Table 4.

As can be seen from Figure 6 and Table 4, the seven groups of small cycle slips can be successfully detected with the proposed algorithm in this paper. The cycle slips on the three frequencies at the 1900th epoch are equal to each other. Taking C25 as an example, it can be found that the test quantities are −0.009 and −0.052, which are not identified by the first two combinations. The combinations are insensitive to the (1,1,1) cycle slip group so that they cannot be detected. The combinations (0,−1,1) and (−1,−5,6) are disabled. However, uniting with geometry-free phase combination can detect the cycle slip successfully. It can be inferred that the combinations may be insensitive to the particular cycle slips, but when the combinations are united, the cycle slips can be detected well. So, the blind zone of detection will be greatly reduced. At the same time, all added cycle slips can be correctly searched and repaired by the minimum L2-norm principle. We can conclude that the inertial-aided cycle slip detection and repair algorithm is capable of detecting and repairing the cycle slips under the condition of small and insensitive cycle slips. Consequently, the availability of the cycle slip detection method proposed in this paper is greatly improved.

**Figure 7:**
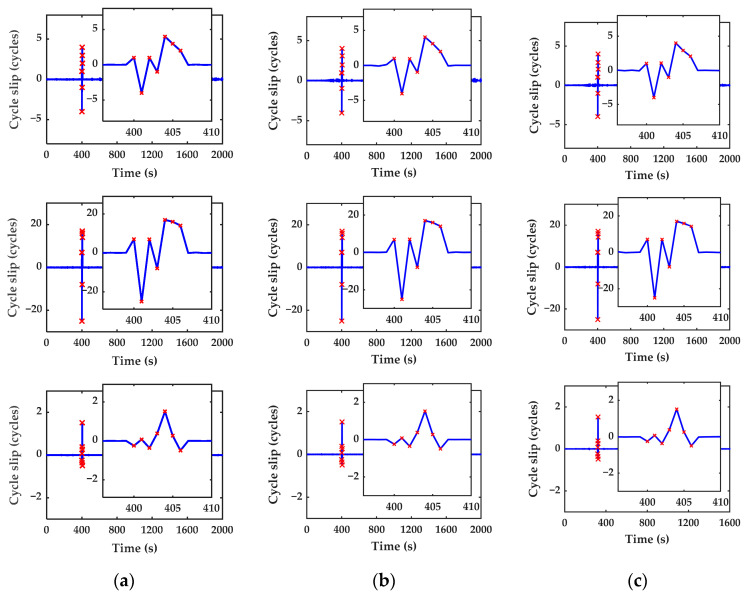
The detection results of the cycle slip simulation (Scheme II): (**a**) C25; (**b**) C39; (**c**) C59.

*Scheme II.* To further test the effectiveness of the proposed cycle slip detection and repair algorithm, the random combination (0,1,2), (3,2,−2), (2,3,4), (2,0,−1), (4,−3,1), (4,2,5), and (0,2,4) which represent intensive and continuous cycle slips, is added to satellite C25, C39, and C59 from the 400th to 406th epochs. The detection and repair results of Scheme II are shown in Figure 7 and Table 5.

It can be clearly found that the test quantities have obviously continuous change from the 400th to 406th epochs. All the manually added cycle slips can be detected and repaired correctly in real time by using the proposed algorithm, as shown in Table 5. So, the inertial-aided cycle slip detection and repair method is not only valid for the small and insensitive cycle slips but also valid for the intensive and continuous cycle slips. The experiment further investigates the effectiveness of the algorithm.

### 4.2. Cycle Slip Detection in BDS Signal Outage Condition

**Figure 8:**
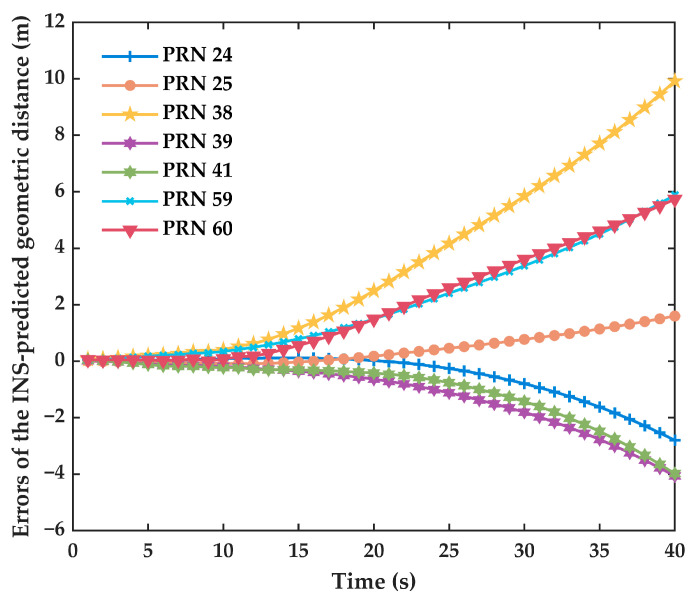
Errors of the INS-predicted geometric distance during the BDS signal outage.

One of the advantages of tightly coupled integrated navigation is that it can still maintain filtering and output navigation information when the signal is disturbed. As a result, it can immediately detect and repair cycle slips when the satellite signal is restored. However, the integrated navigation system will drift slowly during the GNSS signal outage. When attempting to detect and repair the cycle slip, INS-accumulated errors are added into the cycle slip detection value, which may cause incorrect detection and repair of cycle slips. In order to further verify the performance of the proposed algorithm, the BDS signal outage condition was simulated, which was achieved by manually turning off the satellite signal. Meanwhile, to evaluate the detection performance after signal recovery, we added simulated phase cycle slips to all the BDS-3 satellites, except the reference satellite, immediately after the signal is reacquired. During this time, the errors of the INS-predicted geometric distance are shown in Figure 8.

From Figure 8, we can discover that the errors of the INS-predicted geometric distance for satellites are different, but overall, they increase gradually during the BDS signal outage. The INS error accumulates over time, which is constrained by IMU accuracy in the signal outage, but they have the same trend although the accuracy of sensors is diversiform. Additionally, the time limit for correctly repairing the different satellite carrier-phase cycle slips is shown in Table 6. It is defined as follows: if the cycle slip can still be correctly repaired after an interruption of n seconds, then the repairable time limit is considered to be n seconds. The third detection combination is not listed in the table because the INS-predicted value is unnecessary. Consequently, it will not be influenced by the signal outage.

It can be further discovered that to some extent, the trend of errors of the INS-predicted geometric distance in Figure 8 is correlated with the time limit for correctly repairing the cycle slips in Table 6. The errors of the INS-predicted geometric distance for C25 increase slowly, and the time limit for correctly repairing cycle slips is the longest, which is 32 s. In addition, the time limit for correctly repairing cycle slips of the second detection combination is longer than that of the first detection combination, but the final time limit is dependent on the shorter of the two. All the cycle slips can be correctly detected and repaired within a 14 s satellite signal interruption; accordingly, the applicability of cycle slip detection and repair is improved.

## 5. Conclusions

To solve the problem that the traditional cycle slip detection model is easily influenced by the pseudorange observation accuracy, in this paper, an INS-aided cycle slip detection and repair algorithm using double-differenced observations is proposed. The INS-predicted value is constructed to replace the pseudorange and enhance the robustness. The GF phase combination is united to detect the insensitive cycle slip. Furthermore, the optimal coefficient combination is selected, and the L2-norm minimum principle is used to search and confirm the cycle slip repair value. The extended Kalman filter based on the BDS/INS tightly coupled system is established to correct the INS error. Meanwhile, the vehicle experiment was conducted to analyze the performance of the proposed cycle slip detection and repair algorithm. Based on the results and analysis above, we can draw the following conclusions:

(1) The INS-aided cycle slip detection and repair for the BDS triple-frequency algorithm proposed in this paper can be unlimited by the pseudorange observation accuracy. It can complete detection and repair of the cycle slip avoiding the influence of pseudorange noise and a multipath effect.
(2) The proposed algorithm unites the pseudorange-phase combination based on inertial aiding with a geometry-free phase combination. It can be not only valid for the small cycle slips but also valid for the particular case, such as insensitive cycle slips. The results investigate the effectiveness of the proposed algorithm.
(3) A cycle slip repair method based on the L2-norm minimum principle was adopted in this paper, and the experimental results show that all added cycle slips can be correctly searched and repaired by this method.
(4) The cycle slip detection after signal recovery will be impacted by the INS-predicted distance errors. The longer the signal outage time is, the larger the INS-accumulated error is, and the cycle slip detection and repair will be disturbed. All the cycle slips can be correctly detected and repaired within a 14 s satellite signal that is unlocked.

In this paper, we discussed the method of cycle slip detection and repair for the BDS triple-frequency signals, which has certain research value. In the future, further research will be conducted in order to consider refining the cycle slip detection model, theoretically analyzing the influence of the INS uncertainty and their projection on DD and quantitatively analyzing the results of cycle slip detection and repair. At the same time, certain conditions can be set to screen the detected cycle slips to avoid incorrect repairs. The methods can be improved and achieve better results with further exploration and research.

## Figures and Tables

**Table 1:** BDS triple-frequency pseudorange-phase combinations.

*i*	*j*	*k*	*λ*/m	$\eta_{\left(i,j,k\right)}$	$\sigma_{\nabla \Delta \varphi (i,j,k)}$/Cycle
1	4	−5	6.37	0.10	0.13
−1	−5	6	20.93	−0.43	0.16
0	−1	1	4.88	−0.33	0.03
−1	−6	7	3.96	−0.75	0.19
1	3	−4	2.76	−0.22	0.10
1	2	−3	1.77	−0.55	0.07
−1	−7	8	2.19	−1.08	0.21
−3	5	−1	3.57	11.64	0.12
−4	0	5	3.05	11.21	0.13
−4	1	4	8.14	11.54	0.11
−3	6	−2	13.32	11.97	0.14
4	−2	−3	12.21	−11.86	0.11
3	−8	4	2.99	−12.62	0.19
5	3	−9	29.31	−11.44	0.21
5	2	−8	4.19	−11.76	0.19

**Table 2:** BDS triple-frequency geometric-free phase combinations.

*a*	*b*	*c*	$\eta_{(a,b,c)}$	$\sigma_{\nabla \Delta \varphi (a,b,c)}$/Cycle
1	1	−2	−0.36	0.011
1	3	−4	−0.04	0.024
0	−1	1	−0.16	0.007
1	−1	0	−0.68	0.006
1	−2	1	−0.83	0.012
1	2	−3	−0.20	0.018
−3	2	1	1.86	0.016
1	1	−2	−0.36	0.017
2	−1	−1	−1.19	0.010
2	1	−3	−0.87	0.017

**Table 3:** The standard deviation of the cycle slip detection terms for the traditional and improved methods.

Satellite PRN	Elevation Angle (°)	$\sigma_{D}$ (Cycles)		$\sigma_{D-INS}$ (Cycles)	
		(0,−1,1)	(−1,−5,6)	(0,−1,1)	(−1,−5,6)
C24	37~50	0.0711	0.3725	0.0334	0.0860
C25	68~80	0.0510	0.2885	0.0257	0.0662
C38	21~29	0.1453	0.4283	0.0719	0.2022
C39	62~63	0.0506	0.2935	0.0317	0.0689
C41	44~58	0.0613	0.3404	0.0434	0.0765
C59	47~47	0.0522	0.2856	0.0286	0.0796
C60	32~32	0.0642	0.3356	0.0604	0.1352
Average	-	0.0708	0.3349	0.0422	0.1021

**Table 4:** The simulated value and repair result of cycle slips (Scheme I).

Satellite PRN	Epoch	Cycle Slip (Cycles)	Detection Results (Cycles)			$\mathrm{Min}{\left\|A\hat{X\;}-\;L\;\right\|}_{2}$ (Cycles)	Estimated Cycle Slip (Cycles)
			(0,−1,1)	(−1,−5,6)	(1,−1,0)		
C25 (MEO)	100th	(1,0,0)	0.001	−0.993	0.193	0.007	(1,0,0)
	400th	(0,1,0)	−0.985	−4.976	−0.246	0.028	(0,1,0)
	700th	(0,0,1)	0.949	6.039	0.001	0.064	(0,0,1)
	1000th	(1,1,0)	−0.986	−5.908	−0.054	0.093	(1,1,0)
	1300th	(1,0,1)	1.082	5.099	0.192	0.129	(1,0,1)
	1600th	(0,1,1)	0.016	1.072	−0.246	0.074	(0,1,1)
	1900th	(1,1,1)	−0.009	−0.052	−0.056	0.053	(1,1,1)
C39 (IGSO)	100th	(1,0,0)	−0.005	−1.025	0.192	0.026	(1,0,0)
	400th	(0,1,0)	−1.013	−5.052	−0.246	0.054	(0,1,0)
	700th	(0,0,1)	0.971	6.042	−0.001	0.051	(0,0,1)
	1000th	(1,1,0)	−0.996	−5.952	−0.056	0.048	(1,1,0)
	1300th	(1,0,1)	1.010	5.050	0.192	0.051	(1,0,1)
	1600th	(0,1,1)	0.012	1.051	−0.245	0.052	(0,1,1)
	1900th	(1,1,1)	−0.021	−0.121	−0.057	0.123	(1,1,1)
C59 (GEO)	100th	(1,0,0)	−0.004	−1.035	0.193	0.035	(1,0,0)
	400th	(0,1,0)	−0.984	−4.941	−0.246	0.061	(0,1,0)
	700th	(0,0,1)	1.041	5.910	−0.007	0.099	(0,0,1)
	1000th	(1,1,0)	−0.978	−5.902	−0.051	0.101	(1,1,0)
	1300th	(1,0,1)	0.995	4.980	0.189	0.021	(1,0,1)
	1600th	(0,1,1)	0.032	1.172	−0.245	0.175	(0,1,1)
	1900th	(1,1,1)	−0.023	−0.122	−0.060	0.124	(1,1,1)

**Table 5:** The simulated value and repair result of cycle slips (Scheme II).

Satellite PRN	Epoch	Cycle Slip (Cycles)	Detection Results (Cycles)			$\mathrm{Min}{\left\|A\hat{X}-L\right\|}_{2}$ Cycles)	Estimated Cycle Slip (Cycles)
			(0,−1,1)	(−1,−5,6)	(1,−1,0)		
C25 (MEO)	400th	(0,1,2)	1.001	7.007	−0.247	0.007	(0,1,2)
	401th	(3,2,−2)	−3.994	−24.985	0.079	0.016	(3,2,−2)
	402th	(2,3,4)	0.998	6.978	−0.363	0.022	(2,3,4)
	403th	(2,0,−1)	−0.999	−7.997	0.388	0.005	(2,0,−1)
	404th	(4,−3,1)	3.999	17.028	1.509	0.028	(4,−3,1)
	405th	(4,2,5)	2.987	15.986	0.268	0.019	(4,2,5)
	406th	(0,2,4)	2.007	14.019	−0.496	0.020	(0,2,4)
C39 (IGSO)	400th	(0,1,2)	0.987	6.948	−0.246	0.054	(0,1,2)
	401th	(3,2,−2)	−4.033	−25.057	0.078	0.066	(3,2,−2)
	402th	(2,3,4)	0.932	7.047	−0.357	0.083	(2,3,4)
	403th	(2,0,−1)	−0.973	−8.004	0.379	0.028	(2,0,−1)
	404th	(4,−3,1)	4.027	17.046	1.518	0.054	(4,−3,1)
	405th	(4,2,5)	3.091	16.007	0.270	0.091	(4,2,5)
	406th	(0,2,4)	1.972	14.050	−0.497	0.057	(0,2,4)
C59 (GEO)	400th	(0,1,2)	1.016	7.059	−0.246	0.061	(0,1,2)
	401th	(3,2,−2)	−3.983	−25.006	0.080	0.018	(3,2,−2)
	402th	(2,3,4)	1.063	7.024	−0.365	0.068	(2,3,4)
	403th	(2,0,−1)	−1.031	−8.032	0.384	0.045	(2,0,−1)
	404th	(4,−3,1)	4.000	17.018	1.519	0.019	(4,−3,1)
	405th	(4,2,5)	2.903	15.919	0.265	0.127	(4,2,5)
	406th	(0,2,4)	2.072	14.229	−0.492	0.240	(0,2,4)

**Table 6:** Time limit for correctly repairing the different satellite carrier-phase cycle slips.

Satellite PRN	Time Limit (s)		Final Time (s)
	(0,−1,1)	(−1,−5,6)	
C24	21	39	21
C25	32	57	32
C38	16	29	16
C39	18	39	18
C41	14	38	14
C59	17	36	17
C60	22	35	22

## Data Availability

Not applicable.
